# In harm’s way: Non-migration decisions of people at risk of slow-onset coastal hazards in Bangladesh

**DOI:** 10.1007/s13280-021-01552-8

**Published:** 2021-04-06

**Authors:** Bishawjit Mallick, Kimberly G. Rogers, Zakia Sultana

**Affiliations:** 1 CU Population Center Institute of Behavioural Science, University of Colorado Boulder Campus, Boulder, CO 80309 USA; 2 Chair of Environmental Development and Risk Management, Faculty of Environmental Sciences, Technische Universität Dresden, 01217 Dresden, Germany; 3 Integrated Coastal Programs, East Carolina University, 850 NC 345 Wanchese, Greenville, NC 27981 USA; 4 Department of Environmental Science and Disaster Management, Bangabandhu Sheikh Mujibur Rahman Science and Technology University, Gopalganj, 8100 Bangladesh

**Keywords:** Climate, Coastal hazards, Non-migration, Protection Motivation Theory (PMT), Risk perceptions, Social network

## Abstract

Non-migration is an adaptive strategy that has received little attention in environmental migration studies. We explore the leveraging factors of non-migration decisions of communities at risk in coastal Bangladesh, where exposure to both rapid- and slow-onset natural disasters is high. We apply the Protection Motivation Theory (PMT) to empirical data and assess how threat perception and coping appraisal influences migration decisions in farming communities suffering from salinization of cropland. This study consists of data collected through quantitative household surveys (*n* = 200) and semi-structured interviews from four villages in southwest coastal Bangladesh. Results indicate that most respondents are unwilling to migrate, despite better economic conditions and reduced environmental risk in other locations. Land ownership, social connectedness, and household economic strength are the strongest predictors of non-migration decisions. This study is the first to use the PMT to understand migration-related behaviour and the findings are relevant for policy planning in vulnerable regions where exposure to climate-related risks is high but populations are choosing to remain in place.

**Supplementary Information:**

The online version contains supplementary material available at 10.1007/s13280-021-01552-8.

## Introduction

Human migration is a complex behavioural decision shaped by social, economic, demographic, and ecological conditions of the environment within which an individual inhabits (Hunter et al. 2015; McLeman 2018). While not all migration is the result of a strategic choice, the decision to migrate can be considered an adaptive strategy similar to livelihood diversification in the context of adverse environmental conditions or risks (Massey et al. 1998; de Haas 2010; Renaud et al. 2011; Biswas and Mallick 2020). The migration-adaptation discourse (e.g. Warner et al. 2010; Black et al. 2011a; Hunter et al. 2015; McLeman 2018) commonly infers that voluntary migration in response to imminent or actual environmental stress is a strategic decision that reduces climate change risks within a geographic area, particularly in populated coastal regions prone to rapid-onset hazards such as cyclones (Ingram et al. 2006; Groen and Polivka 2010; Mallick and Vogt 2012; Suckall et al. 2017). However, international and domestic migrants collectively comprise less than 16% of the world’s population, suggesting most people remain in place (Rigaud et al. 2018). The cognitive processes behind the decision to stay in hazard-prone areas are likely just as complex as those leading to migration, but there are comparatively fewer studies on the use of voluntary immobility as an adaptive strategy in response to environmental risk (Hjälm 2014; Zickgraf 2018; Bennet et al. 2019; Mallick 2019; Schewel 2019; Mallick and Schanze 2020). Having the aspiration and capacity to remain in place when one is capable of migrating differs from being trapped in a location due to resource constraints or place attachment (e.g. Adams 2016; Nawrotzki and DeWaard 2018). Rather, intentional non-migration is a form of voluntary sedentarism. Sedentarism is commonly understood to be an adaptation that arises from a person’s desire to stay home (Hjälm 2014; Van Hear et al. 2018). In contrast to migration, non-migration can be defined as “spatial continuity in an individual’s centre of gravity over a period of time” (Schewel 2019, p. 329).

Another understudied dimension of environmental migration is the role of slow-onset hazards in the decision to migrate or stay despite these being more globally widespread than rapid-onset events such as cyclones, particularly in low-lying coastal regions. Both ‘slow’ and ‘rapid’ onset refers to the speed of a hazard occurrence, rather than the temporality of the impact of such hazards on affected communities (Montz et al. 2017). Here, we consider cyclones to be rapid-onset phenomena because they form, make landfall, and dissipate within days. By contrast, saltwater intrusion and drought related to climate change are examples of slow-onset environmental hazards that are pervasive in coastal areas but may take months or decades for the cumulative impacts to manifest (Rahman et al. 2019; Hauer et al. 2020). Although the effects of a single cyclonic storm may persist for years, the impacts of a discrete storm event are relatively abrupt compared to the effects of climate-related salinization or drought. Additionally, sediment trapping in channels or reservoirs arising from feedbacks with the built environment can take decades to accumulate before it impacts accretion, erosion, navigation, and water flow in low-lying coastal zones (Giosan et al. 2006; Syvitski et al. 2009). Both slow- and rapid-onset hazards can cause damage or complete loss of cultivatable land, though their onset rate may influence perceptions that contribute to migration decisions (Warner et al. 2010; Renaud et al. 2011).

Overall, the influence of environmental conditions on migration decisions is increasingly acknowledged by migration scholars (e.g. Black et al. 2011b; Gray and Mueller 2012; Chen and Mueller 2018; Cattaneo et al. 2019; Hauer et al. 2020). While these authors link migration to environmental conditions, outstanding questions remain: how do environmental conditions factor into the decision to remain in place? How does the awareness of gradually changing environmental conditions, particularly for rural smallholder farmers who depend on predictable soil and water conditions to maintain their livelihoods, motivate the decision to remain at home? If the same factors influencing migration also guide the decision to remain in place, then non-migration decisions are likewise related to context- and person-specific opportunities associated with livelihood resilience and the (in)capability and aspiration of not migrating. More work is needed to understand the motivations, perceptions, and cognitive processes behind the decision to remain in place, particularly in agriculturally-dominated coastal regions where slow-onset hazards are threatening water and food security (Roy et al. 2016; Chen and Mueller 2018). This study contributes to this knowledge gap through an investigation of the multi-scalar factors contributing to non-migration decisions in agricultural- and fisheries-dominated coastal communities in Bangladesh. We draw on a theory derived from health psychology, the Protection Motivation Theory (PMT) of Rogers (1975) to frame the role of fear and personal appraisal of coping ability in non-migration decisions. We used multi-stage sampling to collect household survey data (*n* = 200) and key informant interviews (*n* = 11) from two districts of coastal Bangladesh where natural and anthropogenic hazards have resulted in soil salinization and in-channel siltation over decadal time scales. The PMT is then applied to examine the social, economic, institutional, and ecological drivers shaping an individual’s cognitive process when the decision to remain in place are made (e.g. Taylor 1999; Mutton and Haque 2004; Chindarkar 2012). This approach allows us to investigate how people view their ability to cope with the risks associated with slow-onset environmental hazards and how these contribute to non-migration decisions.

### Non-migration vs. the opposite of migration

Non-migration is often treated in mobility studies as the contingency or default state when migration is not feasible (Schewel 2019; Mallick and Schanze 2020). Neoclassical theory of migration states that the decision to migrate is the result of a rational calculation of the costs and benefits of moving (Black et al. 2013; Thompson 2017). In this context, non-migration is the outcome when the costs of migrating outweigh the benefits of staying, as though the factors that lead an individual to choose to migrate are simply the inverse for the decision to remain in place. Building on an increasingly structural approach to migration, Massey et al. (1998) argue a component of a cohesive migration theory is the “aspiration” to migrate. Their suggestion provides insight into how non-migration may be understood as an intentional and deliberate behavioural choice, rather than the opposite of migration. Both migration and non-migration can be seen as functions of individual aspiration and capability, where capability refers to the ability to aspire, as well as the ability to realize an aspiration (Carling 2002; Czaika and Vothknecht 2014; Carling and Schewel 2018; Zickgraf 2018). That is, those who aspire to migrate are not always capable of realizing this aspiration. For many, there is no agency involved in the decision to migrate or to stay. Black and Collyer (2014) introduced the term ‘trapped population’ to describe populations who would like to migrate but are incapable of doing so because they have limited economic resources and social connectivity. The distinction between ‘involuntary or forced immobility’ (*aspiration without capability*), selectively being ‘left behind’ while another member of the household migrates (*capability without aspiration*), and ‘voluntary sedentarism’ is that in the latter, both an aspiration and capacity to remain in place are present (Zickgraf 2018; Mallick and Schanze 2020). Other limits and barriers to adaptation through migration arise from psychological views, cultural milieu, and locational disadvantages, resulting in a kind of forced non-migration (Adams 2016; Ayeb-Karlsson et al. 2018). This is distinctly different from intentionally opting to remain in place when the capability to relocate exists, particularly in areas where environmental hazards are commonplace (Mallick et al. 2020; Mallick and Schanze 2020).

An alternative motivation for migration is described by the New Economics of Labour Migration (NELM) theory, which suggests that some members may migrate to benefit the remaining household member that remain in place through the sending of remittances (Stark and Bloom 1985). However, the NELM has been criticized for its limited applicability in non-migration research (de Haas 2010; Abreu 2012). Non-migration also encompasses ‘trans-local livelihoods’, whereby some people within a community temporarily and seasonally migrate, mainly for economic reasons, yet return and therefore remain connected to their origin community (Mallick et al. 2020). In this instance, the members of a household who are ‘left behind’ may benefit from sending a seasonal migrant out of the community to earn money as a way of diversifying the household’s livelihood portfolio (Jónsson 2011; Carrico and Donato 2019). Other theories suggest that the social and cultural milieu of a community are the primary factors holding people to a place, which implies a non-migration decision is related to place attachment rather than the outcome of a rational cost/benefit calculus (Irwin et al. 2004; Adams 2016; Bennet et al. 2019). These theories get closer to framing non-migration as a conscious behavioural choice, yet still present the aspiration to migrate as universal.

## Analytical framework

Scholars have applied diverse theories and conceptual frameworks to understand cognitive behaviour as it relates to migration, including the Stress—Threshold model (Speare 1974), the residential satisfaction model (Speare 1974; Lu 1998), and the theory of planned behaviour (Ajzen 1991; Lu 1998; Speelman et al. 2017). The Stress—Threshold model suggests that in most cases highly satisfied people would not consider migrating despite the fact that they may be better off somewhere else and the decision to migrate is an outcome dependent upon a calculation weighing the costs and benefits of leaving (Wolpert 1966). Similarly, the Residential Satisfaction model explains that intentions of migration or non-migration depend upon residential satisfaction within a community (Speare 1974). The theory of Planned Behaviour describes the relationship between behavioural intention and actual behaviour, particularly the attitudes toward a behaviour, subjective norms, and perceived behavioural control (Ajzen 1991). The theory of Planned Behaviour considers social influence such as social norms and normative belief, but it does not consider how economic and environmental factors may influence an individual’s intention to perform a behaviour, and also does not consider that the behaviour can change over time (Lu 1998). Another theory that could help explain migration behaviour, the Terror Management Theory (TMT), claims that people protect themselves from death anxiety by maintaining their cultural worldviews and self-esteem (Greenberg et al. 1986, 1991). Recently TMT is employed to assess the impact of terrorist incidents on the desire for immigration in European countries (Cruz et al. 2020). However, TMT considers the anxiety-buffering factors (e.g. attachement, self-esteem and meaning of life) for analysing a psychological disorder that hampers the decision-making ability of the individual (Yetzer and Pyszczynski 2018).

In contrast to these cognitive behaviour models, the Protection Motivation Theory (PMT) posits that both anticipated risks and action through either coping or adaptation influence individual cognitive behaviour (Rogers 1975; Grothmann and Patt 2005; Meso et al. 2016). The PMT was first introduced by Rogers (1975) and used in social and health psychology to explain the effect that health communication in the form of fear appeals has on a patient’s choice of treatment. Rogers (1975) claimed that people protect themselves based on: (i) the perceived severity of a threatening event, such as disaster or disease, (ii) the perceived probability of the occurrence of the threatening event, (iii) the efficacy of the recommended preventive behaviour, e.g. belief that adopting a certain behavioural response will be effective in dissipating the threat, and (iv) perceived self-efficacy, e.g. the belief that the individual can successfully perform the coping response. Thus, the basic concept of PMT is that self-protection is achieved through a process that includes appraisal of both the threat and an individual’s ability to cope with it. The PMT was later expanded upon by Maddux and Rogers (1983), Tanner et al. (1989), Floyd et al. (2000), Clubb and Hinkle (2015), and Wong et al. (2016) to include the influence of risk and protection choice perceptions on decision-making. The PMT has since been used to explain behavioural choice in information technology and security (Meso et al. 2016), criminal justice studies (Clubb and Hinkle 2015), and climate change adaptation strategies (see, e.g. Martin et al. 2007; Bubeck et al. 2013; Koerth et al. 2013; Keshavarz and Karami 2016).

**Fig. 1:**
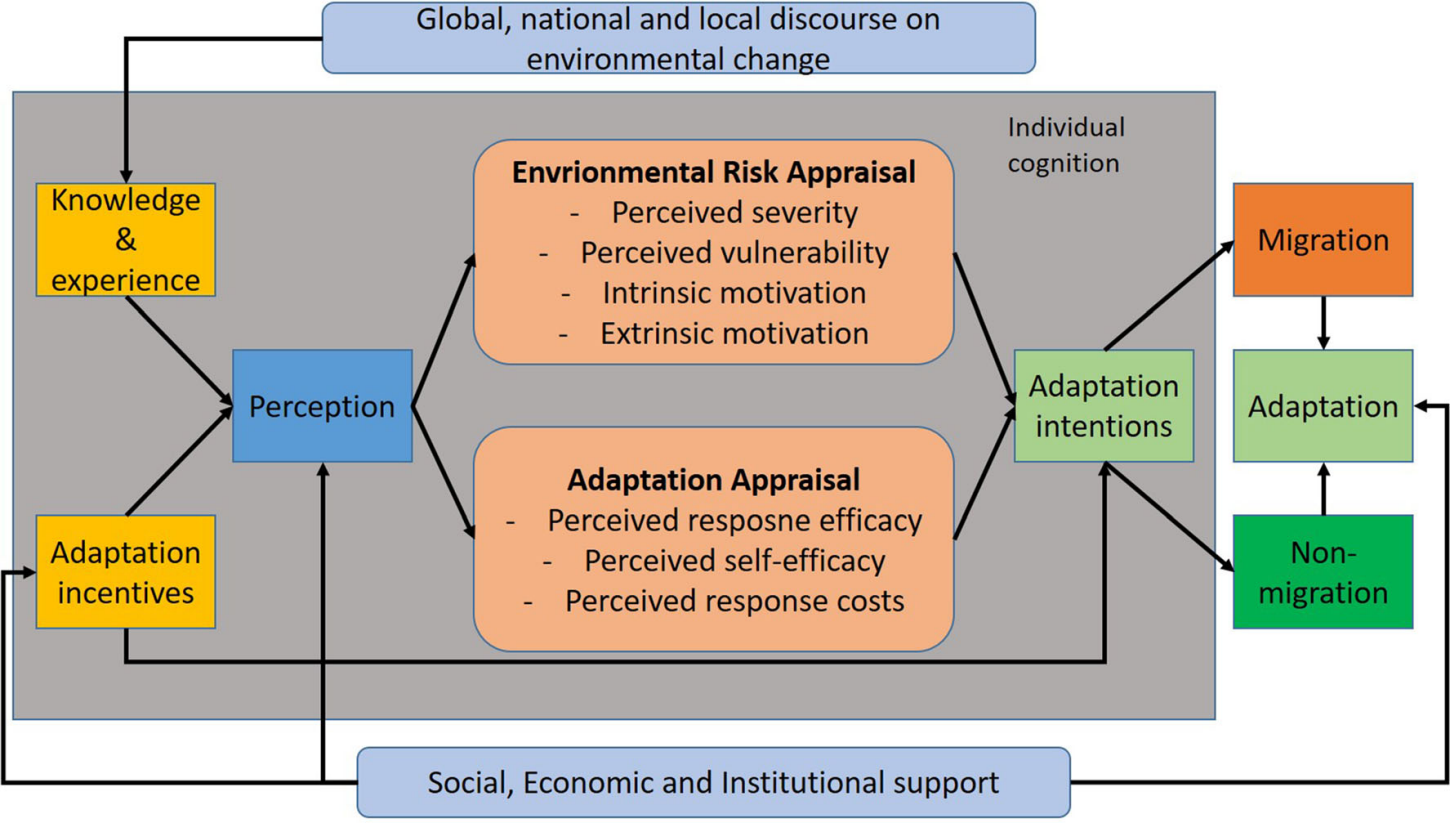
Protection Motivation Theory (PMT) and its constructs, modified from Grothmann and Patt (2005) and Xiao et al. (2014)

According to Rogers’s original theory (1975; Rogers and Mewborn 1976), people facing risk engage in adaptive behaviour through two interlinked cognitive processes: evaluation of the occurrence probability and severity of a threat (i.e. perceived risks), and perception of the effectiveness of a coping response and one’s ability to adopt it (i.e. coping response) (Mishra and Mazumdar 2015). Perceived risks and coping responses are assessed prior to an individual choosing an adaptation strategy. The strategy chosen depends on the skills or resources that an individual has access to, which in turn frames an appraisal of the environmental risk and the potential responses. That is, the PMT consists of two possible techniques that an individual may use to link perception to behaviour, which is not common for other behavioural theories such as the ‘stress-threshold, ‘residential satisfaction’ or ‘planned behaviour’ theories. First, the environmental risks appraisal technique clarifies the severity of and vulnerability to threats, and the intrinsic and extrinsic rewards received from different behavioural responses. Second, the adaptation/response appraisal technique emphasizes the role of individual beliefs in a person’s ability to respond to perceived risks, i.e. self-efficacy, response efficacy, and response costs (Fig. 1). The technique used will depend upon an individual’s experiences, knowledge, social, economic and institutional supports, and demographic characteristics.

Since extreme weather and climatic events increase environmental risk and have the potential to impact people’s living environment, it is assumed that coping and adaptation strategies adopted in response to these are based on risk assessments and resources. In the context of environmental events (both slow and sudden onset) migration or non-migration are adaptation strategies that are embedded in an individual’s risk appraisal and response capacity. Accordingly, the PMT has been used to explain the perceptions of previous flood experience, the risk and perceived ability to cope with future floods, and perceived efficacy and costs of both self-protective behaviour and non-protective responses (Grothmann and Reusswig 2006; Bubeck et al. 2013; Bamberg et al. 2017). In another example, Martin et al. (2007) employed PMT to understand risk-mitigating behaviours undertaken by homeowners in the context of wildfire management. PMT has also been widely used in understanding individual behaviours of people exposed to landslides (e.g. Mertens et al. 2018), drought (e.g. Keshavarz and Karami 2016), and sea-level rise (e.g. Koerth et al. 2013). However, to our knowledge, this is the first time that PMT is applied to explain the role of fear appraisal in non-migration decisions where slow-onset hazards are drivers of environmental migration.

There is a positive relationship between migration intentions and hazard risk perceptions, for example in the case of floods, cyclones and wildfires (Grothmann and Patt 2005; Mallick and Vogt 2014; Nawrotzki et al. 2014; Dorlöchter-Sulser 2015). Here we consider perceptions of slow-onset hazards, i.e. gradual salinization and channel siltation, and how these influence migration decisions in southwest coastal Bangladesh. This area is vulnerable to both rapid-onset and slow-onset environmental events, and inhabitants lack the resources needed to sustain current livelihoods or shift to alternatives. This study includes a cross-sectional analysis of environmental, economic, and social dimensions of migration decisions as guided by the PMT to understand decision-making regarding non-migration.

## Materials and Methods

### Site selection and participants

Previous studies of migration decisions have considered different spatial (internal or international), temporal (short-term or permanent), social (gender, elderly, children, family), economic (employment), and political drivers of migration (Petersen 1958; Abu et al. 2014; Barcus and Shugatai 2018; Zickgraf 2018). Here, a multi-stage sampling approach is used to select 200 households from four villages for individual household interviews. Villages were selected within three unions, the smallest rural administrative unit in Bangladesh, in two coastal districts: Khulna and Satkhira. A union typically contains around 9 individual villages. The four villages selected as study sites were initially identified through reconnaissance interviews. Geographic criteria for site selection were that villages must be located less than a kilometre from a major river or mangrove forest. Other criteria include the presence of widespread livelihood challenges associated with place-specific hazards and a history of socio-political shifts. In this study, we consider socio-political shifts to be an outcome of historical land-use change arising from the shifts in natural resource-based livelihoods, i.e. conversion of rice paddy-land to shrimp farms in the 1980s (see Paul and Vogl 2011). Inclusion criteria for purposively selecting respondents were that they must be at least 18 years of age, self-identifying smallholder farmers owning < 2 ha of land, and able to answer questions related to their individual household as well as the local environment. There were 1948 households in the selected study villages. Considering 95% confidence level and a margin of error of 6.5%, we interviewed 200 households maintaining equal sample distribution in four villages. A detailed description of the sampling criteria is available in the supplementary document. The questionnaire collects the information of a household, therefore our analysis represents the household level information. The rural households in Bangladesh are primarily male-headed; we interviewed female members in the absence of the male-head at home, and thus our analysis reflects the male-dominated perceptions on non-migration decisions. However, this is socio-culturally grounded and acceptable at our specific study sites. Incomplete or implausible data were removed from the study. On average, respondents took 40 min to complete individual surveys. Ethics approval was obtained from Dhaka University of Bangladesh and the respondents were not compensated for their time.

### Environmental setting

**Fig. 2:**
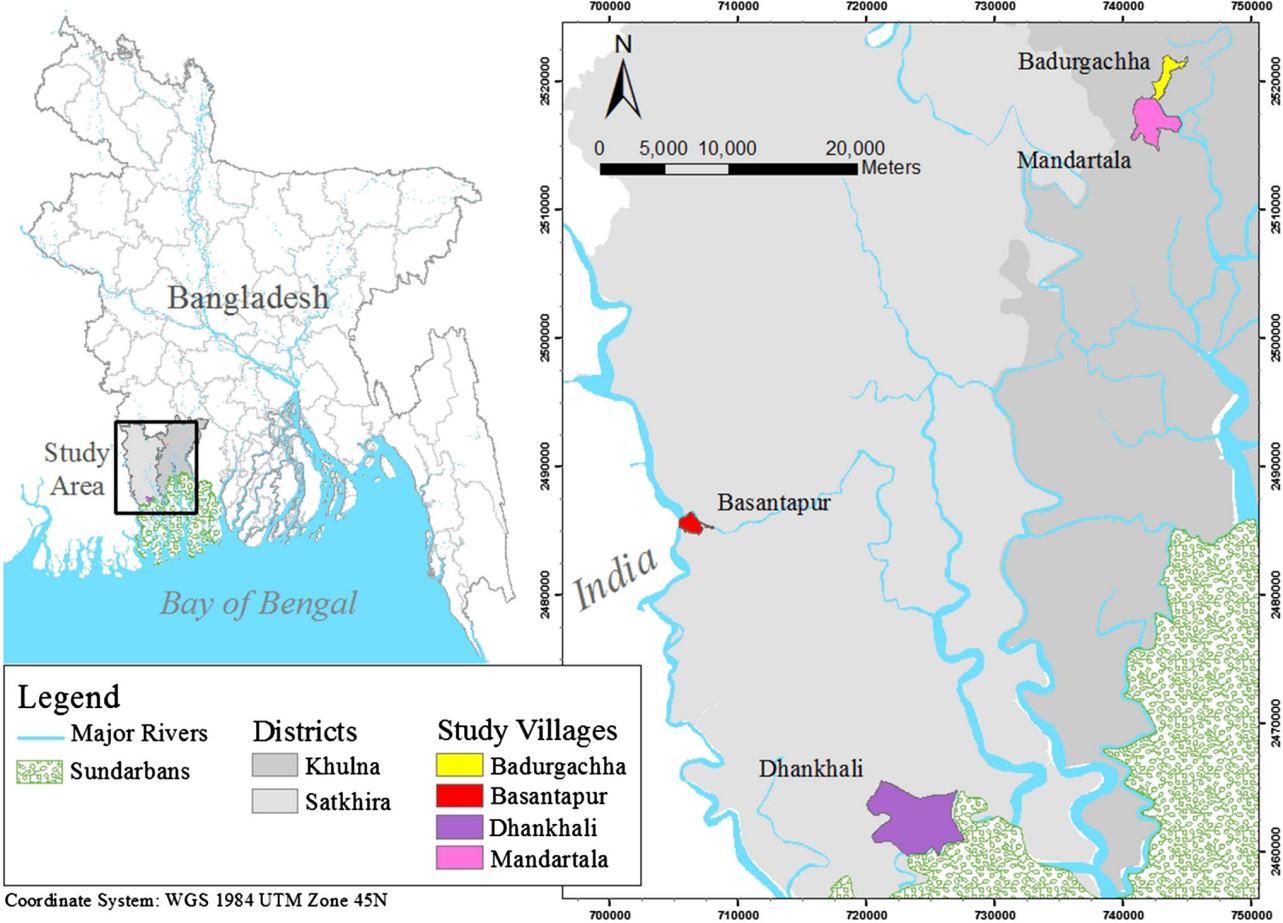
Study area demonstrating proximity to the Sundarbans mangrove forest, rivers, and Bay of Bengal

Household surveys and key informant interviews were conducted at four coastal unions in Khulna and Satkhira districts of southwest Bangladesh (Fig. 2). Both districts lie within a “moribund” area of the delta that receives very limited, if any, freshwater and sediment from upstream river sources (Rogers et al. 2013; Wilson and Goodbred 2015). These districts encompass sections of the Sundarbans Reserve mangrove forest as well as cultivated delta plain that is now used for agriculture and aquaculture. The forest and delta plain are dissected by > 10 000 km of navigable tidal creeks on the order of metres to several kilometres in width. Twice daily meso-tides ranging from 2 to 3.5 m convey sediment-laden water as far as 120 km inland from the coast through this intricate network of channels (PSMSL). While rural farmers have adapted their livelihoods to regular tidal flooding in coastal Bangladesh, soil and surface water salinization has gradually increased throughout the coastal belt since the 1970s (CGIAR 2013). Land outside of the Sundarbans forest was slowly converted from rice cultivation to poorly managed shrimp farming in the 1980s, which accelerated salinization and degraded soils. Farming switched to mixed rice-shrimp production as market demand for rice increased in the early 2000s, though soil fertility remained very low due to the two previous decades of intense shrimp cultivation (Ali 2006). Compounding the deleterious effects of prolonged submergence of land with brackish water for shrimp cultivation was a reduction in freshwater delivery to the coastal region. Natural river migration, construction of the Farakka Barrage on the Ganges River, and subsequent siltation of principal distributaries delivering freshwater to the study area have resulted in reduced dry-season flows, allowing the salinity incursion to penetrate over 100 km inland (Mirza 1998; Winterwerp and Giardino 2012; Shameem et al. 2014; Ayers et al. 2017; Sadik et al. 2017; Wilson et al. 2017).

The proximity of our study sites to the Bay of Bengal also exposes them to storm surges from cyclones that occur an average of once every 3 years (MoEF 2008; Blunden and Arndt 2019). Storm tracks, wind speeds, and timing of landfall within the spring-neap tidal cycle collectively influence the height of storm surge. The extent of saline water intrusion from storm surges magnifies the cost of storms to lives and livelihoods. Cyclones Sidr (2007) and Aila (2009) alone caused an estimated $1.9 billion USD (2009 dollars) in cumulative damage, including crop losses in subsequent years due to waterlogging and persistent salinization of cropland (Khan et al. 2015; Kabir et al. 2016). Approximately one-third of people affected by Cyclone Aila migrated out of the region (e.g. Kartiki 2011; Mallick and Vogt 2012), implying that the majority of people chose to stay despite significant impacts to their livelihoods. Comparatively, approximately 75% of Louisiana’s population remained in place following Hurricane Katrina in 2005 (Groen and Polivka 2008). To protect Bangladesh’s (then East Pakistan’s) agricultural sector from salinity incursions related to coastal flooding, widespread earthen embankments, locally called polders, were built around many inhabited island perimeters outside of the Sundarbans beginning in the 1960s. Embankments were outfitted with sluice gates that could be raised and lowered to allow water to drain from fields at the end of the rainy season. Since their construction, polders have prevented tidal flooding of cropland, but have also restricted the deposition of sediment that normally sustains the elevation of the landscape. Consequently, the interior of poldered islands throughout southwest Bangladesh has compacted while channels outside the polders have silted up, increasing vulnerability to flooding and water logging (Auerbach et al. 2015; Thomas 2020). An estimated 600 km of channels have been disconnected from the main channel network through siltation, creating > 90 km^2^ of new land in the region (Wilson et al. 2017). The few viable channels for delivering river water and enabling navigation are obstructed by shrimp farms and irrigation dams.

### Questionnaire and variables

The questionnaire has ten sections focused on demographic information, livelihood opportunities, migration and non-migration intentions, land-use change, infrastructure management, governance and accountability, environmental hazards, adaptation incentives, and socio-economic conditions. The questionnaire was adapted from the Integrated Social, Environmental and Engineering (ISEE) instrument used by Vanderbilt University and modified for our study (Ackerly et al. 2015; Supplemental Data S1). Previous literature focuses on place attachment and social capital as the main drivers of non-migration (Irwin et al. 2004; Adams 2016; Bennet et al. 2019). We expanded on this to include questions regarding inheritance, wealth and capital resources, and strength of social networks. In this section, we describe the variables we measured and the weight assigned to each.

We measured risk perception from three perspectives. Perceived severity refers to how a respondent perceives relative changes in the salinity of water in the nearest tidal river and canal and to changes in river siltation. Perceived vulnerability refers to how a respondent’s well-being and economic situation has increased, decreased or remained the same due to environmental changes. These two variables (i.e. perceived severity and perceived vulnerability) are measured on a scale from 0 to 2, where 0 means decreased, i.e. perceived no risks, 1 means no change in perception of risk, and 2 means increased, i.e. perceived high risks. Hazard experiences explain the number of events (e.g. salinity intrusion, riverbank erosion, waterlogging, siltation, and cyclone) in the last 5 years that affected the livelihood of both the respondents and their communities. This variable was measured on a scale from 0 to 20. Those who responded all those five types of hazards have impacted their livelihoods and communities receive a score of 20 (5*2*2), and those who responded that nothing has affected their livelihoods and communities receive a score of 0 (zero).

For completing the environmental risk appraisal in our PMT model, we also consider the intrinsic and extrinsic motivations of the respondents that result in a conscious decision to stay, rather than migrate. Intrinsic motivation is a reaction to external variables, such as the local political situation or collective decisions, and acts to either reinforce or diminish the decision to migrate (Winter-Ebmer 1994). In our study, we consider intrinsic motivation to reflect how satisfied respondents are with decisions that affect all community members in their village. It was measured on a scale of 0 to 5, where 0 is never satisfied, and 5 is very satisfied. Extrinsic motivation can influence the decision to migrate or stay and is driven by external rewards, including the respondent’s social status, recognition for good work, or political affiliation. We consider extrinsic motivation to be a function of how influential the person is on community decision-making. It is measured on a scale of 0 to 5, where 0 is not influential at all and 5 is very much influential.

The adaptation appraisal is derived by three significant variables: response efficacy, self-efficacy, and response costs. Risk-taking and handling risks influence migration intentions. People take all kinds of longer-term risks, for example, they invest in land in a peri-urban place, they move to a new location, or they send their son or daughter away for education or work elsewhere. We consider response efficacy as a variable that explains how easy or difficult is it for the respondent to accept taking risks such as these. It is measured on a scale of 0 to 5, where 0 is very difficult and 5 is very easy. Similarly, the self-efficacy variable measures whether the respondent strongly agrees or disagrees that his or her family can survive environmental changes that are affecting livelihood sources. It is also measured on a scale of 0 to 5, where 0 is strongly disagree, and 5 is strongly agree. It is expected that those who agreed that they could manage to shift their livelihood activity due to environmental changes opt for non-migration as an adaptation. Response costs refer to whether the respondent migrated before within or outside their community due to an environmental event and was recorded as either a yes (1) or no (0).

Socio-demographic and economic factors also influence the interplay between the factors of migration decisions following an extreme event (Mallick and Vogt 2014; Nawrotzki et al. 2014). For example, education is usually positively correlated to migration, since better-educated people have a higher chance of making a living outside their community. Hunter et al. (2015) describe that women have a higher risk perception than men and are more likely to migrate internally (e.g. within communities, regions, or cities) in response to environmental hazards, whereas men tend to migrate long-distance (Gray and Mueller 2012; Mueller et al. 2014). Additionally, land ownership and income play a vital role in response to environmental hazards, as studies indicate that landlords and people with greater sources of income do not migrate after hazards, whereas resource-poor people mostly migrate to nearby cities or communities for alternative income opportunities (Kartiki 2011; Mallick and Vogt 2012; Etzold et al. 2015). However, many economically poor individuals are incapable of migrating in response to environmental events because they lack capital or social network strength, or have limitations within the household related to family caregiving (Black et al. 2011b). Taking all of the above into consideration, we included age, education, and income as control variables in addition to those that are relevant to the PMT. We did not consider the gender of the respondent as a control variable, as only 2% of respondents in our sample were female because of the culturally embedded male-domination of household decision-making. Instead, we consider the numbers of female members in the family as a control variable.

### Data analysis

The overall factors motivating non-migration decisions across all of our study sites include place attachment, social network and connectedness, immobile capital (e.g. land ownership, residential house) and economic strength, and access to financial resources and institutions (e.g. credit, savings) (Irwin et al. 2004; Adams 2016; Schewel 2019). However, these factors differ at the individual household level. The dependent variable in our analysis stems from the reasons motivating a respondent to remain in their village and has greater than two response options. Therefore, we employed a multinomial logit model (MNL) to analyse the determinants of the respondents’ intention to remain in place. The response options are of four forms: (i) I have my own land to grow crops and can run my family, e.g. land inheritance and ownership, (ii) My relatives and extended family are living in this village, e.g. social network, (iii) I am economically well-off and can manage any economic crisis in my family, e.g. economic strength, and (iv) others (including permanent employment, engagement with local politics, business, illness of family members, etc.). We used ‘others’ as a response option reference category. The MNL analysis was conducted using SPSS Package Version 22.

Our independent variables are cognitive variables according to the PMT (Table 1) and socio-economic variables are controlled variables. Usually, the parameter estimates of the MNL model provide the direction of the effects of the independent variables on the dependent variable. First, we estimated the correlation among the independent variables to avoid the strong collinearity that may influence the regression results. Second, we ran a baseline model with all independent variables that were selected in the PMT (see Table 1 and Supplementary Table S1 for the variables and Table 2 for the results). We used the Chi square test to explain the relationship between two categorical variables. In addition to this, we employed a post hoc analysis of achieved power of all the PMT variables with respect to the dependent variable. The results derived from G*Power 3.1.9.2 (Faul et al. 2009) show that the power score (1-ß) of the PMT variables lies between 0.82 and 1.0 (see Supplementary Table S3), where the threshold score is 0.8 (Faul et al. 2009; Vadillo et al. 2016).

**Table 1:** PMT variables included in the model and results for individual villages in this study (responses are presented in percentage at village level if not mentioned other). *Source* Field survey 2017

PMT component	Measurement	Level	Overall (*N* = 200)	Badurgacha (*N* = 50)	Basantapur (*N* = 48)	Dhankhali (*N* = 52)	Mandartola (*N* = 50)
Perceived severity -salinity	Has the amount of saline water in the nearest tidal river/canal…?	Decreased (0)	10	12	2.1	9.6	18
		Unchanged (1)	25	22	35.4	26.9	16
		Increased (2)	65	68	62.5	63.5	66
Perceived severity -siltation	Has the siltation in the nearest tidal river/canal…?	Decreased (0)	12	10	10.4	21.2	8
		Unchanged (1)	22	12	14.6	38.5	22
		Increased (2)	65.5	78	75	40.4	70
Perceived vulnerability	How likely do you think your well-being and economic situation has …?	Decreased (0)	11	6	14.6	13.5	10
		Unchanged (1)	62	74	54.2	51.9	68
		Increased (2)	27	20	31.3	34.6	22
Experience (environmental hazards)	In the last five years, how many times has your livelihood and community been affected by any of salinity, erosion, siltation, waterlogging, and cyclone?	Mean (SD)	7.56 (4.81)	7.64 (5.11)	6.25 (5.5)	8.84 (4.65)	7.42 (4.1)
Extrinsic reward	When decisions are made on issues that affect all villagers, do you feel that you are influential in determining the outcome?	Never influential (1)	33	22	43.8	36.5	30
		Rarely influential (2)	21.5	20	25	19.2	22
		Sometimes influential (3)	14.5	14	6.3	15.4	22
		Usually influential (4)	12.5	20	10.4	9.6	10
		Always influential (5)	18.5	24	14.6	19.2	16
Intrinsic reward	Overall, how satisfied are you with the way that the decisions that affect all community members are made in your village?	Not satisfied at all (0)	5.5	12	0	3.8	6
		Not very satisfied (1)	8	4	8.3	7.7	12
		Neither satisfied nor dissatisfied (2)	4.5	4	6.3	5.8	2
		Somewhat satisfied (4)	39	34	35.4	34.6	52
		Very satisfied (5)	43	46	50	48.1	28
Response efficacy	People take all kinds of risks in a year. They borrow money to grow crops/shrimp. They choose to plant a crop they have never grown before. They go for seasonal agriculture in a place they have not been before. In general, how easy or difficult it for you to accept taking risks like these or other risks?	Easy (1)	10.5	14	14.6	5.8	8
		Neither easy nor difficult (2)	32	36	18.8	32.7	40
		Very difficult (3)	57.5	50	66.7	61.5	52
Self-efficacy	The environment is changing and affecting your sources of livelihood, but your family can survive such changes, please state your opinion on it.	Strongly disagree (1)	8.5	6	12.5	11.5	4
		Somewhat disagree (2)	13.5	8	22.9	15.4	8
		Neither agree nor disagree (3)	19.5	24	16.7	19.2	18
		Somewhat agree (4)	26	24	18.8	25	36
		Strongly agree (5)	32.5	38	29.2	28.8	34
Response cost	Have you ever moved your whole household temporarily to another place within this village because of an environmental event?	No (0)	64.5	86	45.8	61.5	64
		Yes (1)	35.5	14	54.2	38.5	36

**Table 2:** Multinomial model results (*N* = 200)

Predictors	Reason-1: Land inheritance and ownership		Reason-2: Social network		Reason-3: Wealth and capital strength	
	B	OR	B	OR	B	OR
Intercept	.929		− 1.419		− 3.240	
Age of the household head	.030	1.031	.054**	1.055	.032	1.033
Income of the household	.000	1.000	.000	1.000	.100***	1.000
Number of female members in HH	.761	2.141	1.029***	2.799	1.295***	3.653
Level of education of household head						
Illiterate	− .194	.824	− .260	.771	− 1.453	.234
Less than 10 years of schooling but not illiterate	− 1.965	.140	− 2.608***	.074	− 3.448***	.032
More than 10 years of schooling^a^						
Hazard experience	− .054	.947	− .130	.878	.026	1.026
Risk tolerance						
Easy	− 2.521**	.080	− 1.777	.169	− 1.533	.216
Neither easy nor difficult	− 1.389	.249	− .996	.370	− 1.414	.243
Difficult^a^						
Self-efficacy						
Strongly disagree	20.070***	1.508	20.804	1.3567	20.290	1.27829
Somewhat disagree	2.451	11.601	1.581	4.858	2.922**	18.576
Neither agree nor disagree	1.544	4.683	.490	1.632	.762	2.143
Somewhat agree	.393	1.482	.985	2.679	1.650	5.206
Strongly agree^a^						
Perceived vulnerability						
Decreased	.100	1.105	− 1.357	.258	.442	1.555
Remained the same as before	− 1.072	.342	− .755	.470	.661	1.937
Increased^a^						
Response cost (Yes = 1; No = 0)	1.600***	4.955	1.081	2.947	1.388	4.007
Perceived severity: Saline Water						
Decreased	− 1.909**	.148	− .225	.798	− .042	.959
Remained the same as before	− 1.842	.158	− 1.745**	.175	− 1.308	.270
Increased^a^						
Perceived severity: (River Siltation)						
Decreased	− 1.571	.208	− 2.575***	.076	− 3.362***	.035
Remained the same as before	− .636	.529	− 1.551**	.212	− .971	.379
Increased^a^						
Intrinsic motivation						
Not satisfied at all	.824	2.280	− .014	.986	.385	1.469
Somewhat satisfied	1.658	5.247	2.177	8.816	− 17.506	2.497E − 08
Neither satisfied or dissatisfied	21.290	1.89223	21.010	2.4566	22.699	1.278299
Somewhat satisfied	.870	2.387	1.702	5.487	1.351	3.862
Very satisfied^a^						
Extrinsic motivation						
Never influential	− 1.016	.362	.375	1.455	− .640	.527
Rarely influential	− .472	.624	.925	2.522	.211	1.235
Sometimes influential	− 2.933***	.053	− 1.785	.168	− 2.860***	.057
Usually influential	− .901	.406	− 2.024	.132	− 1.244	.288
Always influential^a^						
Pseudo R2	0.495					
Log likelihood	377.394					
LR chi2	136.483					

**p < 0.1, ***p < 0.05

^a^Indicates the reference category of the independent variables; “Others reasons” of non-migration is the reference category of the dependent variable

## Results

In the following section, we describe the quantitative demographic characteristics of our study areas and determinants needed for conducting the PMT analysis. Qualitative information collected through key informant interviews has been interspersed to provide context to our quantitative results.

### Sample characteristics

Detailed demographic results from each village are presented in Table S1 and key results are summarized here. Overall, the mean age of respondents across all study sites is 49.3 years. Mean household female members in our sampling frame are 2.3, whereas the mean number of household members is 4.7. The overall religious identity of respondents is 35% Muslim and 65% Hindu. While half of our study villages contain a mixture of both, the other two are comprised of entirely one or the other. That is, 100% of respondents in Badurgacha village in Shovna Union are Hindu, while Muslims constitute 95.8% of respondents in Basantapur village in Mathurespur Union. The distribution of religious identity in our study does not reflect that of all of Bangladesh, which as of the 2011 census was reported at 89% Muslim and 9% Hindu (BBS 2012). The share of Muslim and Hindu population in Khluna district is 77.6% and 22.4%, respectively, whereas in the Satkhira district, the share is 81.8% and 18.2%, respectively. This offset is due to the selection procedure of the study sites. That is, our selection criteria were based on geographic proximity to rivers or tidal channels and therefore the study villages experience livelihood challenges related to location-specific hazards and have a history of land-use change, i.e. widespread conversion of rice paddy-land to shrimp farms. Adult literacy across our study sites averages 76%, which is similar with the country’s overall literacy rate of 74% for adults over 15 years of age in 2018 (MRDI 2020). The highest percentage of illiterate respondents in our study villages were in Basantapur (35.4%) and the lowest in Badurgacha (10%). Mean income is highest in Mandartala village in Shovna Union (~ $230 USD per month) and lowest in Basantapur village (~ $204 USD per month). Individual respondents in Badurgacha own more land (average ~ 1.5 ha) compared to other study sites and respondents of Dhankhali village in Munshiganj Union owned the least (average ~ 0.3 ha). Quality of housing materials is also the lowest in Dhankali, which is very near to the Sundarbans forest boundary. Respondents conveyed that this is due to high soil salinity and lack of quality building materials. Locally available materials such as Golpata wood is harvested from nearby mangroves and used to construct houses. The settlement history question reveals that a majority of the respondents’ family (60.5%) has lived in their community since before their grandfather, whereas 10.5% since their grandfather, 14.5% since their father, and the remaining 14.5% of the respondents were the first members of their family to move into their present community. These results do not significantly influence the non-migration motivation (Chi square = 15.433, *p* < 0.199).

With regard to the motivation behind decisions to remain in place, land ownership and its related farming opportunity are reported as the main reasons for non-migration (41.5%) followed by land inheritance and strong local social ties (27.5%) (i.e. the respondent has relatives and extended family members living in the same village), economic strength (20%), and others (including permanent employment, engagement with local politics, business, illness of family members, etc.). These results do not significantly differ across the four study villages.

### Description of the PMT determinants

An underlying premise of the PMT is that self-protection is achieved through a process that includes the appraisal of a threat and an individual’s perception of their ability to cope with that threat. Here, we first describe our respondent’s perceptions of slow-onset hazards in their area, specifically soil and water salinization and channel siltation, and then present results related to the factors influencing beliefs in their ability to self-manage these gradual environmental changes. Over 60% of the respondents in three of our four study villages perceives that salinity of the water in the nearest tidal river or canal has increased in the 5 years prior to our study. The only village where salinity is reported as “high” but unchanged over the previous 5 years is Basantapur, located in Mathurespur Union along the India-Bangladesh border. This is likely due to Basantapur’s location adjacent to a large tidal channel and lack of connection with freshwater river distributaries. Respondents here also described “water scarce” conditions. They report that due to saline groundwater, their drinking water comes solely from ponds and year-round cultivation of rice is not possible. Therefore, rice crops are only grown during the rainy season. Several respondents ascribe the area’s saline soil conditions to the vast conversion of land to saltwater shrimp farms that began in Satkhira District in the 1980s (Akber et al. 2017). Another source of soil salinity is cyclones and strong coastal storms, though the impact of storms is highly localized. For example, all respondents that we interviewed in Munshiganj Union described they were unable to produce crops for up to 3 years due to soil salinization following Cyclone Aila in 2009. Yet interviewees in Mathurespur Union claimed they were not impacted by Aila’s storm surge and crop productivity was unaffected, though the two unions are less than 30 km apart. Despite this spatial variability, background salinity levels in southwest Bangladesh are greatly enhanced in any area following a storm surge.

In addition to increased salinity, infilling of tidal channels and canals by siltation is an ongoing phenomenon in this region of Bangladesh. According to Wilson et al. (2017) an estimated 600 km of formerly viable channels have been clogged with sediment and cut off from the channel network. This is due to both natural and anthropogenic processes. Extensive poldering in the region has disconnected secondary channels from larger tidal rivers, thus reducing the overall volume of water that can be transported through the system. Reduced channel network connectivity and diminished velocity of sediment-laden tides result in enhanced sedimentation within channels that over time emerges from the channel bed as new land, or “khashland” (Wilson et al. 2017). Specific to our study sites, an average of 65.5% of respondents across three of four study villages report that siltation within the nearest tidal river or canal has increased. However, most inhabitants of Dhankhali village report they are living on khashland that has been in place for decades. One 42-year old respondent described this:

I saw in my childhood that there was a slope from the land to the canal and the canal had higher depth of water than the agricultural lands. But you cannot see that now; the canal has been totally silted in but has (only) a slight slope there from the lands.

The perception that channel siltation has not changed in the 5 years before our study implies that these former canals have all reached sedimentation capacity and have been under cultivation for years. In addition to reduced network connectivity, embankment sluice gates that are typically raised and lowered to allow monsoon floodwater to drain off cropland have become clogged with silt and are no longer functioning. This effectually closes off internal drainage canals from the tidal channel network, perpetuating siltation within perimeter channels.

These slow-onset changes in the environment have impacted the economic state of the farmers in our study. We hypothesized that those whose economic conditions have improved would be the least likely to migrate. In our study sites, only 12.5% reported that their well-being and economic standing had increased over the previous 5 years. Only 2.8% of respondents reported that their well-being and economic situation had decreased whereas most (84.7%) report stable conditions. This suggests that although most of our respondents detect the slow-onset environmental changes happening in their area, they perceive these changes have not negatively impacted their ability to make a living. Mounting risk through stacked hazards may play a role in non-migration decisions (e.g. Black et al. 2011b). As the location of our study sites exposes them to hazards beyond siltation and salinization, i.e. erosion, waterlogging, and cyclones, we asked our respondents about their overall experiences with cumulative hazards. Our study shows that in the last 5 years, the respondents in all villages faced an average of eight hazards (7.6), with those in Dhankahli reporting nine hazards (8.8), and people in Basantapur reporting six hazards (6.3). We expect that respondents who have experienced fewer hazards would be more likely to remain in place.

Being respected and responsible for the community influences perception at the individual level (Bonjour and Chauvin 2018). We found that almost one-third of respondents (33%) felt that they were not influential in community-level decisions that affect all villagers. However, almost half of all respondents were satisfied with the way that decisions affecting all community members in their village were made. Significant variations in the responses regarding both these extrinsic and intrinsic motivations across study villages were not found.

With regard to risk-taking, almost half of all respondents reported that it is challenging for them to take risks such as borrowing money to grow crops or shrimp, planting a crop that they have not planted before, or temporarily migrating for seasonal agricultural work in a place they had not worked before. However, over half of all respondents agreed that their family could survive changes in the environment that are affecting their livelihood sources. It seems risk tolerance also influences future migration decisions. An average of 35.5% of respondents within three of the four villages had moved their whole household temporarily to another place within their villages because of an environmental event. Except the people living in Badurgacha, only 14% of the respondents in their village were displaced due to an environmental event. Across all of our sites, 64.5% of respondents had never been displaced, even within their community, due to an environmental event.

### Non-migration decisions

**Fig. 3:**
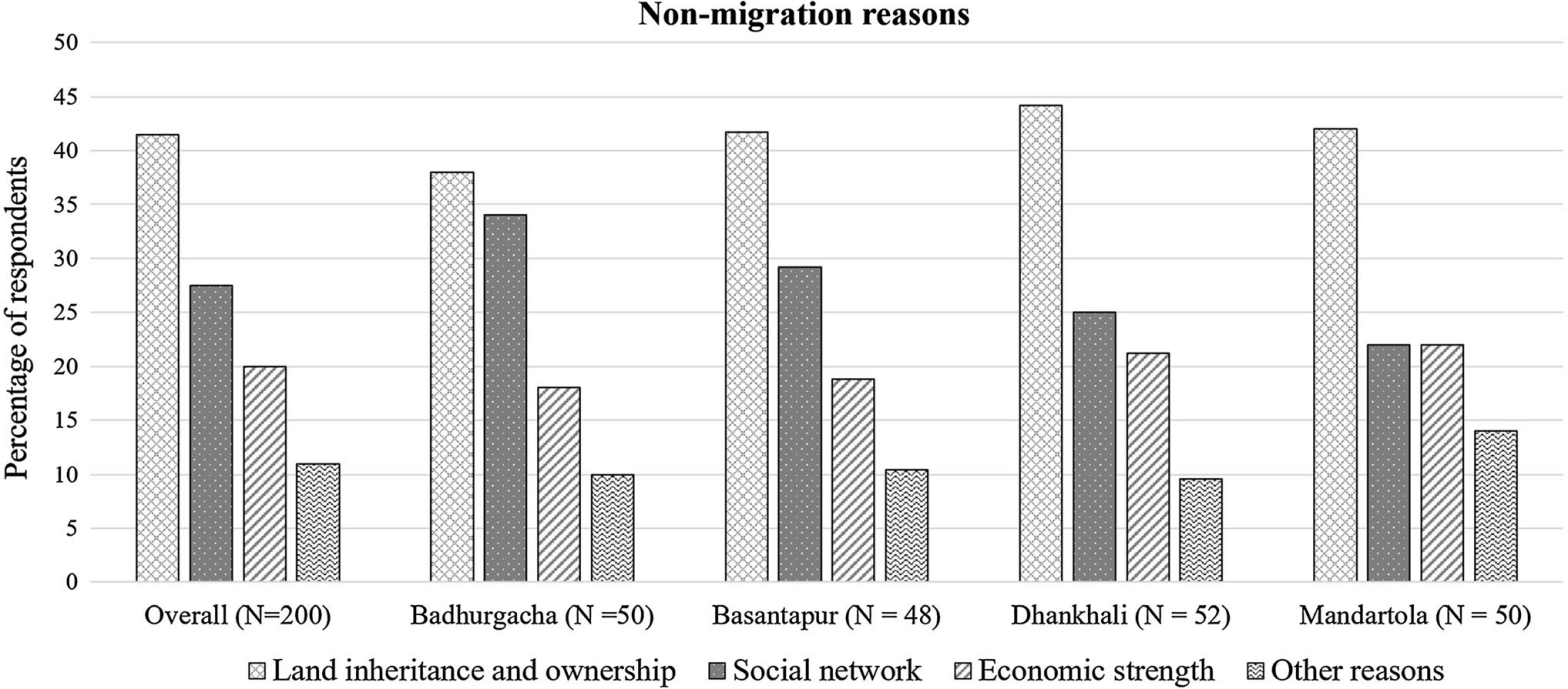
Non-migration reasons from 2017 field survey

The respondents reported land inheritance and ownership as the prime reasons for their non-migration decisions, followed by the strength of their social networks, and wealth and capital (Fig. 3). “Land inheritance and ownership” used here means that respondents own the title to the land where they grow rice and other crops. In Badhurgacha and Basantapur villages, people prefer to be connected with their relatives and extended family members (“social network”) rather than be economically solvent. In contrast, results from Mandartola village show that social network and wealth and capital strengths are equally important in non-migration decisions. The overall distribution of these reasons across the study sites are not statistically significant (Chi square = 12.5, *p* < 0.799), and therefore, in this section, we investigate the variations in the reasoning behind respondents’ non-migration decisions, i.e. how and to what extent different variables influence non-migration aspirations of the people in these communities.

### Determinants of non-migration decisions

We conducted a multinomial regression (MNL) to explore the influence of different independent variables on reasons given for non-migration decisions. Table 2 shows the results of the MNL. Our regression model indicates that age is an important indicator for those who claimed “social network” to be one of the primary and positively correlated reasons for remaining in place, i.e. older people prefer to stay. Similarly, respondents with more female members in the family reported both social network and economic strength as the reasons behind their non-migration decisions. Respondents having less than 10 years of schooling were less likely to migrate compared to those with more than 10 years of schooling. As expected, respondents with a higher income chose “economic strength” as the primary reason behind their non-migration decisions. However, no demographic factors showed significant influence on “land inheritance and ownership” as the driver of non-migration.

#### Land inheritance and ownership

Five PMT variables are shown to influence “land inheritance and ownership” reasons for non-migration: two environmental risk appraisal variables, i.e. perceived severity and extrinsic motivation, and three adaptation appraisal variables, i.e. self-efficacy, response efficacy and response cost. Those who possess land want to grow crops, principally rice so that they can feed their family. In this context, salinization of soil or water can impact a respondent’s ability to produce crops on their land. Our model shows that a perceived decrease of salinity in the river and soil increases the selection of “land inheritance and ownership” as the main driver of non-migration decisions. If the salinity levels in the river and canal (i.e. perceived severity) were to decrease by 1% compared to increasing by the same amount, the multinomial log-odds of selecting “land inheritance and ownership” for non-migration would increase by 83.2% with all other variables in the model held constant.

The social acceptance of people in the village-level society of Bangladesh is related to the amount of their landholdings and wealth (Mallick 2014). Individuals who are particularly involved in various social activities (e.g. campaign and relief work, cultural activities, volunteering) within a community are less likely to want to migrate. In most cases, they are the landlords and wealthy people of the community, as societal acceptance in rural Bangladesh is commonly inherited through landlord-ships known as Zamindars. In other words, those who feel that they are influential in their community would prefer to remain in place (Mallick and Vogt 2014). Thus, extrinsic motivation plays a role in “land inheritance and ownership” determining a decision to remain in place. Our model results indicate that a 1% increase in the perception of not being influential compared to those who perceive they are always influential (i.e. when decisions are made on the issues that affect all villagers), the multinomial log-odds of selecting “land inheritance and ownership” relative to other reasons for non-migration are expected to decrease by 47% with all other variables held constant. In other words, “land inheritance and ownership” contribute to being influential in the community-level decision-making process, and therefore it promotes non-migration.

All three adaptation appraisal variables, i.e. response efficacy, self-efficacy and response cost, have significant influences on “land inheritance and ownership” as a driver of non-migration decisions. Results show that increased risk tolerance reduces the selection of “land inheritance and ownership” as the primary reason for choosing to remain in place. For example, a 1% increase in the ability of a respondent to more easily take risks (i.e. response efficacy) results in a 20% decrease in the multinomial log-odds of selecting “land inheritance and ownership” as the primary reason behind a non-migration decision. Similarly, increased disagreement on survival despite changes in livelihood as a result of environmental change (i.e. self-efficacy) increases the selection of “land inheritance and ownership” as the primary reason behind opting to not migrate. For example, a 1% increase in the disagreement on the ability to survive despite threats to livelihood-making due to environmental changes (self-efficacy) results in a 50% decrease in the multinomial log-odds of selecting “land inheritance and ownership” as the primary reason behind non-migration. Again, having any sort of migration experience (response cost) reduces the chance of choosing “land inheritance and ownership” as the primary reason for opting to remain in place. Thus, our result shows that respondents who had previously never migrated are almost five times more likely to choose “land inheritance and ownership” as the primary reason behind non-migration than those who had migrated before. However, being affected by hazards does not have a significant influence on “land inheritance and ownership” for non-migration decisions.

#### Social network

Two PMT variables, perceived severity of siltation and self-efficacy, have significant relevance on opting for “social network” as the reason for a non-migration decision. The perceived severity of siltation variable relates to the siltation conditions in the nearest tidal river or canal. Our model shows that decreased siltation in the most adjacent river and canals increases the selection of “social network” as the primary reason for choosing to remain in place. For example, a 1% decrease of river or canal siltation results in a 92.4% increase in the multinomial log-odds of selecting “social network” as the primary reason behind a non-migration decision. We interpret this to mean that decreasing siltation in nearby rivers or canals leads to increased fishing and water resource-based economic opportunities at the local level, which requires social connections and cooperation in order to be successful (Curran 2002). Again, our model shows that there is a significant causal relationship between the agreement of a respondent’s ability to survive despite threats to livelihood-making due to environmental changes (self-efficacy) and choosing “social network” as the primary reason for non-migration. Our results also show that a 1% disagreement on the ability to survive despite threats to livelihood-making due to environmental changes results in 65% decrease in the multinomial log-odds of selecting the chance of “social network” as the primary reason for non-migration decisions. In other words, interdependency between people living in a community is the key to long-term non-migration.

In addition to the PMT variables, three demographic variables (age and education level of the respondent, and number of female members in the household) also showed significant influence on selecting “social network” as the reason for non-migration. The results indicate that a 1-year increase in the respondent’s age results in a 5.4% increase in the chance of choosing “social network” as the reason for non-migration. In other words, the older someone is, the more likely they are to claim that their social network keeps them rooted to a place. However, the number of female members in the family also significantly influences “social network” as the primary motivator of a non-migration decisions. For example, the results show that an increase of one female member in the family results in a 179.9% increase in the multinomial log-odds of selecting “social network” as a primary reason for non-migration. Females are more likely than their male counter parts to maintain social networks (e.g. Szell and Thurner 2013), implying that the number of female family members is an important indicator of whether the decision to stay or migrate is taken. Furthermore, the cultural and religious orientation of Bangladesh towards protecting females also influences the decision to migrate. If a respondent has a comparatively high number of female family members, they are likely to consider the safety and social security of these family members when deciding to migrate or relocate (Ayeb-Karlsson 2020). Similarly, the years of schooling of the respondent has a significant causal influence on selecting “social network” as the primary reason for non-migration. For example, an individual with a minimum of 10 years of schooling is 26% less likely to choose “social network” as the primary reason for non-migration than an illiterate individual.

#### Economic strength

Two PMT variables, i.e. perceived severity of siltation and extrinsic motivation, have a significant causal relationship to selecting “economic strength” as a primary reason for non-migration. If siltation in the adjacent rivers and canals increases, then the chance of choosing “economic strength” as the reason for non-migration decreases. For example, a 1% increase in siltation in the adjacent rivers and canals results in a 65% decrease in the multinomial log-odds of selecting the “economic strength” as the primary reason for non-migration. This indicates that increased siltation of the nearest rivers and canals may create water scarcity for crop production, and thus affect the economic strength of the individual household. With respect to an individual’s extrinsic motivation, here defined as the role an individual plays in the community decision-making process, a 1% increase in the perception of not being influential when the community decisions are made results in a 43% decrease in the multinomial log-odds of selecting “economic strength” as the reason for non-migration. In other words, people who influence local decisions are also most likely to be economically solvent, and therefore prefer to stay. At the individual household level income plays a minor role in “economic strength” being the main reason for non-migration. A 1% increase in an individual’s household income results in a 10% increase in the multinomial log-odds of selecting “economic strength” as the reason for non-migration. In other words, income solvency helps a respondent remain in place. Conversely, the number of females in the household significantly influences the chance of “economic strength” being the primary reason for non-migration. Adding one more female member to a household relative to a household with no female members results in a 265.5% increase in the multinomial log-odds of selecting “economic strength’ as the reason behind a non-migration decision. This primarily indicates the economic importance of marriage, education, and social security of the girls in the family. Demanding dowry for a bride is common in rural communities in Bangladesh, so if a family has a daughter, the parents should save money/resources for arranging the marriage. Finally, the education variable explains that people who have never gone to school relative to those who have more than 10 years of schooling are 76.6% less likely to choose “economic strength” as the primary reason for non-migration. That is, our model confirms that the more educated a respondent is, the more they claim that their economic strength is the reason for staying.

## Discussion

The PMT offers several advantages to understanding how perceptions of both risk and an individual’s capacity to respond to threat factors into non-migration decisions. First, the PMT presents a single model for explaining the relative contributions that perceived risks and adaptive capacity indicators have on individual-level decisions on migration; there is no other analytical model that provides this for non-migration decision analysis. Second, the PMT also allows for the exploration of the “reasons behind the reasoning”, i.e. it enables more in-depth analyses by examining two levels of reasoning. In this study, the PMT allowed the identification of the individual-level factors behind each specific reason given for not migrating. The risk appraisal factors explain the importance of slow-onset environmental changes in non-migration decisions. For example, our results indicate that an individual landowner believes they can still produce crops if there is a perceived decrease in channel salinization; therefore, they will choose to remain in place. In contrast, the adaptation appraisal factors suggest that the individual-level response efficacy, self-efficacy, and response costs have considerably more influence on future non-migration decisions.

Overall, our results suggest there are three main reasons people at risk of slow-onset hazards choose to remain in place rather than migrate: (i) land inheritance and ownership, (ii) the strength of their social network and their personal influence, and (iii) economic strength. All three reasons are related to the concept of place attachment (Adams 2016). Although settlement history is also connected with land ownership, assets, and strength of social and economic conditions (e.g. Zhou et al. 2013), we do not see a significant association between settlement history and non-migration. This may be related to site selection criteria, in that we selected sites relative to their proximity to channels and their land-use history. Several changes in socio-economic conditions have been reported due to conversion from rice to shrimp, and thereafter, these respondents have chosen to remain in place.

**Fig. 4:**
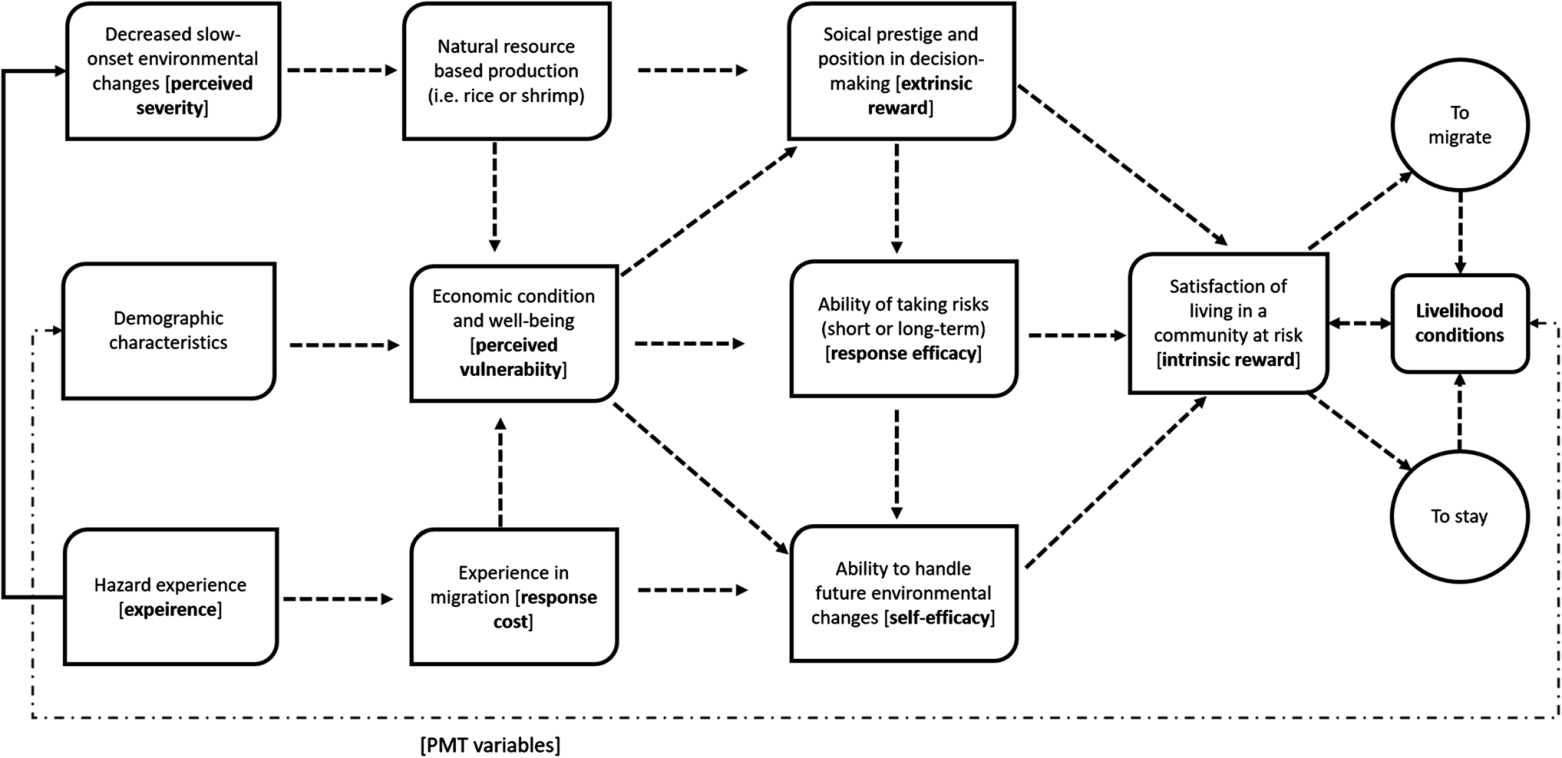
Causal interrelationship among PMT variables that influence non-migration decision

The most notable result is the influence of land inheritance and ownership on non-migration decisions, which is the only outcome represented equally across all demographic categories (Table 2). The causal relationships between the variables used in the PMT model are demonstrated in Fig. 4. In this causal network, migration and non-migration are outcomes of the level of satisfaction of living in a community at risk of slow-onset environmental hazards, i.e. the intrinsic reward governs the decision to migrate or to stay. Both non-migration and migration contribute to local livelihood conditions. In the case of non-migration, people who stay in the village exploit nearby natural resources or seek work-for-hire near their village, e.g. collecting shrimp fries from the river to sell at the market, day labouring for cash (including maintaining shrimp farms), or cultivating vegetables in village courtyards to sell during the rainy season when soil salinity is decreased. Individuals who migrate, particularly seasonal migrants who go to cities or internationally to find work, do not depend on local resources but support village livelihoods through the sending of remittances. Economic conditions, which is a perceived vulnerability factor of our PMT model, influence the ability of an individual to self-manage future environmentally-related changes in their livelihood. The PMT model refers to this as self-efficacy and effects the satisfaction of living in a place. Similarly, economic conditions frame the social prestige and public image of an individual household, and therefore this acts as an extrinsic motivation that influences the overall satisfaction of living in a place.

The experience of being affected by natural hazards is not significant for any of the three reasons for non-migration. This suggests environmental hazards do not necessarily motivate non-migration decisions, but may contribute to the experience of migration (Fig. 4). People who have had experience with both past environmental hazards and migration perceive that they are capable of surviving future hazards, demonstrating self-efficacy. We found that extrinsic motivation, i.e. motivation driven by external rewards such as positive social status, recognition for good work, or political affiliation has a significant influence on non-migration choices (Fig. 4). Perceived or actual changes in siltation and salinity in the nearest tidal rivers and canals also have a significant influence on the decision to stay home as these impact agriculture and fisheries production in the study villages. Future studies can expand on this work by including the effects of environmental recovery lag times on non-migration decisions, that is, factoring in the time that it takes for land to be suitable for crop production following salinization. This would enhance our understanding of the thresholds between non-migration and migration in rural hazard-prone coastal environments.

The study has a few constraints. First, the sample size of the study was relatively small (*n* = 200) and geographically constrained to coastal Bangladesh. This may raise questions regarding the broad representativeness of the findings, particularly with regard to statistical treatments. As we applied a purposive multi-stage sampling approach that considered different geographical and socio-political attributes in selecting our study villages, our claims may be representative for coastal Bangladesh more broadly. Further empirical research using a larger sample size collected from a wider range of geographical settings is needed to evaluate and validate the contribution of PMT to understanding voluntary non-migration decisions. However, to our knowledge, this is the first study to demonstrate that land inheritance and ownership, social status, and network connectivity play a role in an individual’s decision to remain in place despite risks posed by gradual environmental change. Second, a gender bias is reflected in our very small sample number of female respondents and therefore obscures household power dynamics and the role of females in decision-making. However, our findings are representative for Bangladesh where rural communities are culturally and socially patriarchal. Finally, a disadvantage of employing the PMT is that it does not consider the impact of social norms arising from, e.g. religious beliefs, and therefore continued work will be needed to understand the role of informal institutions and cultural influences beyond which a non-migration decision becomes a migration decision. That is, at what level does environmental risk become so great that land ownership and social status are overridden and no longer provide an individual with a sense of self-efficacy? The results provide insight to the importance of individual-level social influence on the formation of novel community-level social norms related to environmental migration. As sea level continues to rise and dam and embankment building continues in watersheds and coastal zones, salinization and shifts in sediment dispersal will become more widespread in low-lying coastal areas. This work gives insight on how individuals currently make the decision to remain in place despite perceptible and potentially livelihood-threatening slow-onset hazards, and provides a baseline for examining how decision-making evolves in response to environmental change. Local-scale adaptive capacity building and planned relocation efforts in hazard-prone coastal areas will be most effective when the relationship between constantly evolving environmental change and behavioural decisions is better understood.

## Supplementary Information

Below is the link to the electronic supplementary material.

Electronic supplementary material 1 (PDF 259 kb)
