# Supplementary material for: In harm’s way: Non-migration decisions of people at risk of slow-onset coastal hazards in Bangladesh
Source: Ambio. 2021 Apr 6;51(1):114–34. doi: 10.1007/s13280-021-01552-8 (PMC8651874; doi:10.1007/s13280-021-01552-8)
Supplement: Supplementary file 1 — Electronic supplementary material 1 (PDF 259 kb) [file 13280_2021_1552_MOESM1_ESM.pdf]

Ambio
Electronic Supplementary Material
This supplementary material has not been peer reviewed

In harm's way: Non-migration decisions of people at risk of slow-onset coastal hazards in Bangladesh

Bishawjit Mallick, Kimberly M. Rogers, Zakia Sultana

Sampling procedures

We employed a multi-stage sampling method as described below:

- 1. Geographic criteria: candidate village must be located within one kilometre of a significant river or mangrove forest.
- 2. Socio-politcal criteria: candidate village has wide-spread livelihood challenges associated with place-specific hazards and a history of socio-political shifts. This includes historical land-use changes in natural resource-based livelihoods, e.g., land conversion from agriculture to aquaculture, climate-related stressors such as exposure to coastal storms, etc.
- 3. Vocation and land ownership criteria: purposively selected small-holder farmers, defined as owning less than 2 hectares of land and more than 18 years old so that he/she was able to answer questions related to their households .

Sample calculation

According to the Bangladesh Bureau of Statistics’ population census of 2011, there were 187, 887, 641 and 243 households lived in Badurgacha, Basantapur, Dhankhali and Mandartola villages, respectively. Considering 1948 total households, we calculated the sample size as 204 at 95% significance level and a margin of error of 6.5%. The sample size (n) is calculated according to the formula:

n = [z2 * p * (1 - p) / e2] / [1 + (z2 * p * (1 - p) / (e2 * N))]

Where: z = 1.96 for a confidence level (α) of 95%, p = proportion (expressed as a decimal), N = population size, e = margin of error.

z = 1.96, p = 0.5, N = 1948, e = 0.065

n = [1.962 * 0.5 * (1 - 0.5) / 0.0652] / [1 + (1.962 * 0.5 * (1 - 0.5) / (0.0652 * 1948))]

n = 227.3136 / 1.1167 = 203.56

n ≈ 204

The sample size (with finite population correction) is equal to 204.

Using a similar method, we also calculated individual village level samples, resulting in 570 samples required for a total of 1948 households. This sample distribution produces the following: for Badurgacha 103 against 187, Basantapur 181 against 887, Dhankhali 168 against 641, and Mandartola 118 against 243 total households. Due to time, limited access to study sites, and budgetary restrictions, we considered 204 samples and distributed them equally across the sites.

Table S-1: Socio-demographic results

Demographic factors	Measurement	Overall (200)	Badurgacha (50)	Basantapur (48)	Dhankhali (52)	Mandartola (50)
Age	(Mean, SD) in years	49.3(12.6)	51.67 (15.04)	48.52 (11.38)	46.7 (11.57)	50.38 (12.0)
Gender ration	Female members in household (mean)	2.29(1.04)	2.24(0.89)	2.47 (1.09)	2.34 (1.06)	2.1 (1.1)
Religion	Muslim (%)	35	0	95.8	40.4	6
	Hindu (%)	65	100	4.2	59.6	94
Level of education	Illiterate (%)	24	10	35.4	26.9	24
	Less than 10 years of schooling (%)	47	42	50	51.9	44
	More than 10 years of schooling (%)	29	48	14.6	21.2	32
Level of income	(Mean, SD) in BDT	18250 (11320)	17465 (9760.85)	17382.3 (11632.4)	18605.86 (11573)	19497.7 (12363.98)
Land asset	(Mean, SD) in Decimal	193.2 (306.6)	371 (407)	103.74 (202.23)	73.78 (93.18)	225.55 (334.09)
Housing condition	Cyclone tolerant (%)	23.5	24	37.5	7.7	26
	Not cyclone tolerant (%)	76.5	76	62.5	92.3	74
Reasons for non-migration	I have my land to grow crops/rice and can run my family (%)	41.5	38	41.7	44.2	42
	My relatives and extended family are living in this village (%)	27.5	34	29.2	25	22
	I am economically well-off and can manage any economic crisis in my family (%)	20	18	18.8	21.2	22
	Others reasons (%)	11	10	10.4	9.6	14

Source: Field survey 2017

Table-S2: Settlement history

6.1 When did your people first come to this village?	1.2 Study village (percentage of the respondents)				Total
	Badurgacha	Basantapur	Dhankhali	Mandartola	
We moved our HH here	12	8.33	28.85	8	15
My father (if female, my husband's father) moved his family here	10	12.50	15.38	20	15
My grandfather (if female, my husband's grandfather) moved his family here	18	2.08	3.85	18	11
My family (if female, my husband's family) has been here since before my grandfather	60	77.08	51.92	54	61

Source: Field survey 2017

Table-S3: Post-hoc analysis of achieved power with respect to the dependent variable

	Reasons for non-migration (DV)	Self-efficacy	Perceived vulnerability	Response cost	Extrinsic reward	Intrinsic reward	Response efficacy	Perceived severity – salinity	Perceived severity – Siltation
Sample (N)	200	200	200	200	200	200	200	200	200
Mean	2.0050	2.3650	1.1600	0.3550	1.7650	2.6850	2.4150	1.5500	1.5300
Std. Deviation	1.02970	0.82168	0.59681	0.47971	0.89655	0.69872	0.87556	0.67063	0.70824
Effect size		0.386	0.97	2.04	0.254	0.783	0.438	0.506	0.531
α err prob		0.05	0.05	0.05	0.05	0.05	0.05	0.05	0.05
Power (1-β err prob)		0.9711	1	1	0.813	1	0.992	0.991	0.99

Note: For power analysis, we have used G*Power 3.1.9.2 software, and found that all the independent variables have at least 80 percent chance of correctly explaining the model's outcome, as the power is above 0.8.
